# Functional Characterization of VDACs in Grape and Its Putative Role in Response to Pathogen Stress

**DOI:** 10.3389/fpls.2021.670505

**Published:** 2021-06-16

**Authors:** Tengfei Xu, Xiaowei Wang, Hui Ma, Li Su, Wenyuan Wang, Jiangfei Meng, Yan Xu

**Affiliations:** ^1^State Key Laboratory of Crop Stress Biology for Arid Areas, College of Horticulture, Northwest A&F University, Yangling, China; ^2^College of Horticulture, Hebei Agricultural University, Baoding, China; ^3^College of Enology, Northwest A&F University, Yangling, China

**Keywords:** *Plasmopara viticola*, *Vitis*, reactive oxygen species, voltage-dependent anion channels, *VpVDAC3*

## Abstract

Voltage-dependent anion channels (VDACs) are the most abundant proteins in the mitochondrial outer membranes of all eukaryotic cells. They participate in mitochondrial energy metabolism, mitochondria-mediated apoptosis, and cell growth and reproduction. Here, the chromosomal localizations, gene structure, conserved domains, and phylogenetic relationships were analyzed. The amino acid sequences of VDACs were found to be highly conserved. The tissue-specific transcript analysis from transcriptome data and qRT-PCR demonstrated that grapevine VDACs might play an important role in plant growth and development. It was also speculated that VDAC3 might be a regulator of modulated leaf and berry development as the expression patterns during these developmental stages are up-regulated. Further, we screened the role of all grape *VDACs*’ response to pathogen stress and found that *VDAC3* from downy mildew *Plasmopara viticola*-resistant Chinese wild grapevine species *Vitis piasezkii* “Liuba-8” had a higher expression than the downy mildew susceptible species *Vitis vinifera* cv. “Thompson Seedless” after inoculation with *P. viticola.* Overexpression of *VpVDAC3* resulted in increased resistance to pathogens, which was found to prevent VpVDAC3 protein accumulation through protein post-transcriptional regulation. Taken together, these data indicate that *VpVDAC3* plays a role in *P. viticola* defense and provides the evidence with which to understand the mechanism of grape response to pathogen stress.

## Introduction

Grape is one of the most important fruit crops worldwide. Most grape lines are derived from parental or ancestral *Vitis vinifera* which are highly susceptible to pathogens that cause serious pre- and post-harvest diseases. These infections have negative effects on grape quality, yield, production, processing, and export. Downy mildew is a common and destructive systemic grape infection caused by the biotrophic oomycete *Plasmopara viticola* (Wong et al., 2001). It is the foremost threat to grape production and can destroy vineyards in most grape-growing regions of the world (Vercesi et al., 1999).

Hypersensitive response (HR) is a type of programmed cell death (PCD) where in reactive oxygen species (ROS) such as O^2–^ and H_2_O_2_ accumulate, which is characterized by DNA fragmentation and chromatin condensation and clustering along nuclear membranes (Levine et al., 1996; Wang et al., 1996). In addition, HR is also used as a defense mechanism triggered by several pathogens, such as bacteria, fungi, insects, and viruses (Wei et al., 1992), which is manifested by rapid curtailing of water and nutrient supply to the affected tissue in the attempt to prevent pathogen proliferation and contact with healthy cells, therefore, it is also a typical feature of immune responses in plants (Dangl and Jones, 2001; Lam et al., 2001). Zamzami (1995) found that mitochondria exhibited reduced transmembrane potential and ROS accumulation before initiating the cell death biochemical reactions, and alteration in mitochondrial permeability is an early event in apoptosis and occurs before nuclear DNA fragmentation, chromatin condensation, and other biochemical processes that culminate in PCD (Kroemer et al., 1997). Genes in mitochondria regulating HRs may also participate in various apoptotic pathways (Johal et al., 1995; Greenberg, 1996).

Voltage-dependent anion channels (VDACs) are some of the most abundant and well-studied proteins in the mitochondrial outer membrane. They were first reported in paramecia and participates in transport across mitochondrial outer membranes (Mayer et al., 1993; Robert et al., 2012). VDACs may also control mitochondrial and cellular energy conversion as well as mitochondrial outer membrane permeability (Colombini, 2004; Young et al., 2007). VDACs may also interact with mitochondrial and cytoplasmic proteins and regulate several mitochondria-related resistance responses (Fiek et al., 1982; Cheng et al., 2003). Hence, VDACs may play complex roles in cell physiology and PCD.

Plant *VDACs* comprise a small multigene family that might be involved in plant stress responses by regulating cell death (Vianello et al., 2012; Takahashi and Tateda, 2013). A previous study showed that overexpression of rice *OsVDAC4* induces apoptosis by a mechanism resembling that of human *HVDAC1* (Godbole et al., 2013). Thus, it triggers PCD in mammalian cells (Godbole et al., 2013). PCD was detected in tobacco BY2 cells and leaves overexpressing *OsVDAC4* and in rice overexpressing *Pennisetum glaucum VDAC* (Desai et al., 2006; Godbole et al., 2013). VDAC transcript and protein were upregulated in response to biotic and/or abiotic stress and mediated PCD (Benz, 1994; Lacomme and Santa Cruz, 1999; Desai et al., 2006; Lee et al., 2009; Tateda et al., 2009). Several *Arabidopsis* VDACs were implicated in biotic stress and upregulated in response to *Pseudomonas syringae* pv. tomato DC3000 (*Pst* DC3000) (Robert et al., 2012). However, there is no clear evidence of the biological function of the VDAC family in grape or its response to pathogen stress.

The aim of this study was to identify *VDACs* in the grapevine genome, and to analyze the biological function of VDAC proteins in response to pathogen stress in grapevine. The results of our study are intended to lay a theoretical foundation for further construction of the regulation network of *VDACs* in grape and other fruit trees, and provide candidate gene resources for creating a new grape germplasm that is high in quality and disease resistant.

## Materials and Methods

### Plant Materials

“Thompson Seedless” (*V. vinifera*) and “Liuba-8” (*Vitis piasezkii*) were obtained from the grapevine germplasm orchard at Northwest A&F University, Yangling, Shaanxi, China. The plants were cultivated under natural environmental conditions and conventional management. *Nicotiana benthamiana* plants were grown in a greenhouse at 25°C daytime and 20°C nighttime. *Arabidopsis thaliana* ecotype Columbia-0 (Col-0; wild-type) was raised in a growth chamber at 22°C, 70% RH, 16/8 h light/dark.

### Bioinformatic Analysis of VDACs

The mitochondrial porin signature (MPS) motif regions of VDACs were aligned using the DNAMAN 9.0 software. PyMOL was used to visualize the three-dimensional structures of the VvVDACs protein.

### Phylogenetic Analysis and Gene Structures of VDACs in Plants

Phylogenetic characterization of VDACs was performed using MEGA 7.0 with the default options (Kumar et al., 2016). Phylogenetic trees were constructed using the maximum likelihood method. The bootstrap values of 1000 replications were performed, keeping the other parameters as the default. GSDS (Gene structure display server) V2.0^1^ was employed to identify the gene structure of VvVDAC genes.

### RNA Isolation, RT-PCR, and qRT-PCR

Total RNA was extracted with an E.Z.N.A.^®^ plant RNA kit (Omega Bio-tek, Norcross, GA, United States). First-strand cDNA was synthesized from 1 μg total RNA in 20 μL reaction mixture using a Prime Script RT reagent kit (Takara Bio Inc., Kusatsu, Shiga, Japan). Real-time PCR (RT-PCR) was conducted in a PCR detection system (Bio-Rad Laboratories, Hercules, CA, United States). Quantitative real-time PCR (qRT-PCR) was performed in the IQ5 real-time PCR system (Bio-Rad Laboratories, Hercules, CA, United States) with SYBR Green qRT-PCR Super Mix (Transgen Biotech, Beijing, China) and using standard protocols. The thermal profile was 95°C for 5 min followed by 45 cycles of 95°C for 10 s, 58°C for 30 s, and 72°C for 10 s. The standard curve was used to calculate relative transcript quantities. Gene expression was calculated relative to *Actin* as the internal reference. The qRT-PCR primers are listed in Supplementary Table 1.

### Expression Profiles of *VvVDACs* in Different Organs and Tissues

For the expression profile of *VvVDAC* genes, we utilized the RNA-seq data from National Center for Biotechnology Information (NCBI) gene expression omnibus (GEO) datasets with series entry GSE36128^2^ (Fasoli et al., 2012), which obtained from a *V. vinifera* cv “Corvina” (clone48) in various organs and tissues at different developmental stages. Heat maps were generated using Heatmap Illustrator, version 1.0.

### Pathogen Strains and Inoculations

*Plasmopara viticola* was maintained on *V. vinifera* “Thompson Seedless” and prepared as a sporangial suspension (Xu et al., 2014). The inoculation was performed according to the reconstruction method of Liu et al. (2015).

*Pseudomonas syringae* pv. tomato DC3000 was cultivated in King’s B (KB) liquid medium supplemented with 25 μg mL^–1^ rifampin at 28°C for 48 h. The bacteria were collected and resuspended in infiltration buffer containing 10 mM MgCl_2_ and Silwet L-77 at OD_600_ = 0.002. Either bacterial suspension or 10 mM MgCl_2_ (mock-inoculation control) was infiltrated with a 1 mL needleless syringe into the laminae of 4-week-old transgenic and wild-type plants. Inoculated plants were then covered with a transparent plastic film for 3 days and photographed (Canon EOS 200D II, Japan).

### Transient *N. benthamiana* Leaf Transformation

Full-length cDNA corresponding to *VpVDAC* was cloned into the binary vector pCambia 1307 containing 3FLAG tags driven by the 35S promoter. The recombinant plasmids were introduced into *Agrobacterium tumefaciens* strain GV3101. The latter was then used in transient *N. benthamiana* transformation by the leaf infiltration method (Goodin et al., 2008). GV3101 cultures bore the construct p35S:VpVDAC or pCambia 1307-3FLAG (empty vector; negative control). The proapoptotic positive control Bax (Lacomme and Santa Cruz, 1999) and the RNA silencing inhibitor P19 were also cultured. All cultures were grown until OD_600_ ∼1.0. They were then centrifuged at 5000 × *g* for 10 min and their supernatants were discarded. The pellets were resuspended in acetosyringone (AS) medium [10 mM MES (pH 5.7), 10 mM MgCl_2_, and 150 μM acetosyringone]. OD_600_ was then adjusted to 0.8 and working suspensions were prepared by mixing all four strains with equal volumes. The mixed suspensions were incubated for 1 h at 22°C and infiltrated into *N. benthamiana* leaves with a needleless syringe. The plants were transferred to a greenhouse for 1 day and moved to a dark room at 22°C for 2 days until 3,3′-diaminobenzidine (DAB) analysis (Thordal-Christensen et al., 1997). The stained leaves were transferred to a destaining solution consisting of acetic acid:glycerol:ethanol (1:1:3, v/v/v) in a water bath at 95°C for 10 min, then kept in 70% ethanol until imaging (Canon EOS 200D II, Japan) was performed.

### Transient Grapevine Leaf Transformation and *Plasmopara viticola* Infection

The coding sequence of *VpVDAC3* was cloned into the pCambia 2300 binary vector containing YFP driven by the 35S promoter. The recombinant plasmid and pCambia 2300 empty vector were introduced into *A. tumefaciens* strain GV3101. A transient transformation was performed *via* vacuum infiltration. Three weeks old plants were filled with bacterial suspensions [10 mM MES (pH 5.7), 10 mM MgCl_2_, and 150 μM acetosyringone] with OD_600_ = 0.8 and placed in a vacuum chamber attached to a circulating water vacuum pump (Model#SHZ-D, Shanghai, China) and exposed to four 20 min vacuum/5 min vacuum release periods (0.08 MPa) to aid plant uptake of the suspension. The infiltrated explants were co-cultivated for 12 h at 22°C and a relative humidity of 80% in darkness, then transferred to a regular 16-h-light/8-h-dark cycle. They were then induced after 24 h *via* spraying with 50 mM β-estradiol and 0.1% Tween. The agro-infiltrated leaves were then inoculated with *P. viticola*. After 1 day post-inoculation (dpi), 4 dpi, and 6 dpi, leaf discs were punched out of the transformed leaf areas, and H_2_O_2_ was assayed using a fluorimetric hydrogen peroxide assay kit (Sigma-Aldrich, MAK165-1KT, United States) following the protocols provided by the manufacturer. The final fluorescence of the experimental group was obtained by deducting the measured fluorescence of the control group from the measured fluorescence of the experimental group. The average sporangia density of *P. viticola* in two different genotypes at 6 dpi (hours post-inoculation) was calculated.

### *Arabidopsis* Transformation

The *A. tumefaciens* floral dip transformation method was used for generating VpVDAC3 overexpressing lines (Clough and Bent, 1998). pCambia 2300 binary vector containing p35S:VpVDAC3-YFP and pCambia 2300 empty vector were transformed into the Col-0 background. T3- or T4-generation homozygous plants were used for phenotype analysis.

### Protein Extraction and Immunoblot Analysis

The leaves were excised, flash-frozen in liquid nitrogen, and pulverized. Total protein was extracted with denaturing buffer [4 M urea, 65 mM Tris–HCl (pH 7.3), 3% (v/v) sodium dodecyl sulfate (SDS), 10% (v/v) glycerol, 0.05% (w/v) bromophenol blue, and 10 mM dithiothreitol (DTT)]. The protein concentrations in the supernatants were determined with a bicinchoninic acid (BCA) protein assay kit (CW0014; CWBiotech, Beijing, China). The proteins were loaded onto 10% SDS-PAGE and transferred to polyvinylidene fluoride (PVDF) membranes which were then incubated with a monoclonal α-GFP antibody (Roche; Cat-No: 11814460001, monoclonal, mouse, dilution 1:2000) and a FLAG antibody (Sigma-Aldrich; Cat-No: F4042, monoclonal, mouse, dilution 1:3000). Anti-mouse horseradish peroxidase (HRP) secondary antibody (Abcam, Cat-No: ab205719, dilution 1:10,000) was used for detection. The PVDF membranes were then incubated in Ponceau S solution [40% (v/v) methanol, 15% (v/v) acetic acid, and 0.25% (w/v) Ponceau S] and destained with deionized water. Images were captured with ChemiDoc^TM^ XRS 170-8070 (Bio-Rad Laboratories, Hercules, CA, United States).

### Plant Damage and Histochemical Assays

Leaves were stained with DAB to investigate H_2_O_2_ accumulation. The leaves were excised, incubated in 1 mg mL^–1^ DAB (Sigma-Aldrich Corp., St. Louis, MO, United States) in the dark overnight, and destained with 95% (w/v) ethanol until clear. Images were then captured under a microscope (Olympus BX−51, Tokyo, Japan).

Cell death was visualized by trypan blue staining. Inoculated leaves were soaked in 0.125 mg mL^–1^ trypan blue solution [phenol/lactic acid/glycerol/water (1:1:1:1)], boiled in a water bath for 2 min, left at room temperature overnight, destained with 95% (w/v) ethanol, and observed under a light microscope (Olympus BX−51, Tokyo, Japan).

### Electrolyte Leakage

Six round infiltrated leaf disks (1 cm in diameter) were excised from each infiltrated leaf, washed in ddH_2_O for 30 min, and placed in test tubes each containing 10 mL water. Solution conductivity was measured with a digital conductivity meter (Leici Corporation, China). Electrolyte leakage was measured at 0, 6, 12, 24, and 48 h. Data are representative of three independent experiments.

### Availability of Data and Material

Sequence data are cataloged in the NCBI database under the following accession numbers: AtVDAC1 (At3g01280), AtVDAC2 (At5g67500), AtVDAC3 (At5g15090), AtVDAC4 (At5g57490), and AtVDAC5 (At3g49920).

### Statistical Analysis

Data are representative of three independent experiments. The data were processed in SPSS v. 17.0 for Windows (IBM Corp., Armonk, NY, United States). One-way analysis of variance was used to determine the significance of the differences among treatment means. Error bars indicate standard deviation (SD) and significant differences are indicated with “^∗^” (*P* < 0.05) or “^∗∗^” (*P* < 0.01). Graphs were plotted with OriginPro 2015 (OriginLab, Northampton, MA, United States).

## Results

### Mapping the VDAC Family in *V. vinifera*

All *VvVDAC* genes were mapped to island cotton chromosomes based on genome annotations. The annotation revealed that the six grape *VDAC* (*VvVDAC1–VvVDAC6*) and one variant were dispersed on five chromosomes (Supplementary Table 2). And *VvVDAC1, VvVDAC4*, and *VvVDAC5* located in chromosome 1, 11, and 14, respectively. *VvVDAC2* and *VvVDAC3* located in chromosome 7. *VvVDAC6.1* and its splice variant *VvVDAC6.2* were located on chromosome 17. The members of this alternative splicing pair differed by 18 amino acids.

Phylogenetic analysis of VvVDACs from *V. vinifera* showed that all VvVDACs were clustered into five distinct categories. VvVDAC1 and VvVDAC5 were clustered together, and the other three members (VvVDAC2, VvVDAC3, and VvVDAC4) also aligned together (Supplementary Figure 1). VvVDAC6 clustered separately from the other five members. To further understand the structural evolution of the *VvVDAC* genes, these genes were subjected to conduct the exon–intron organization (Supplementary Figure 1). Most VvVDAC family members contained five intron and six exons, while the VvVDAC4 had one more intron.

Cluster analysis of the VvVDAC amino acid sequences showed that full length of the grapevine VvVDAC gene family is highly diverse (Supplementary Table 3). Amino acid sequence alignment disclosed that the VvVDAC family members shared 7.6–91.7% similarity. The VvVDAC2 shared least similarity with other VvVDACs.

### 3D Structure Prediction and MPS Analysis of the VDAC Family in *V. vinifera*

**FIGURE 1:**
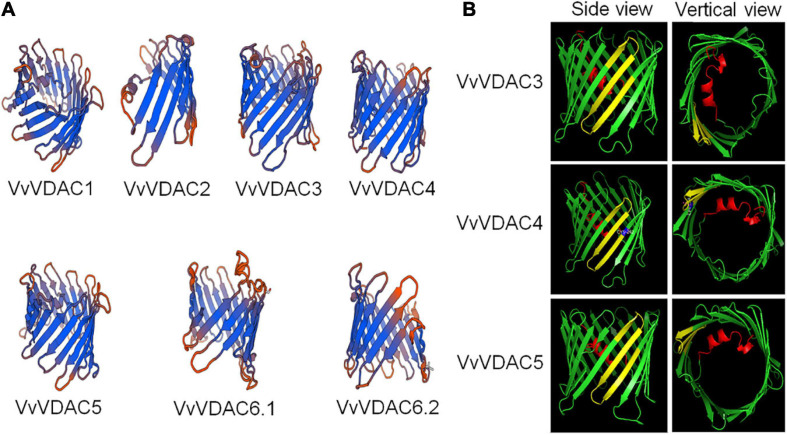
Three-dimensional structure prediction and MPS domain localization of *VvVDACS*. **(A)** Three-dimensional structure of *VvVDACS* is represented by a ribbon diagram. **(B)** Prediction of MPS motif structures for *VvVDAC3*, *VvVDAC4*, and *VvVDAC5* (side and vertical views). *VvVDAC* is a β-barrel (in green) formed by anti-parallel β-strands; the strands are connected by loops (in green). The N-terminal domain (in red) is arranged in an α-helix and is located inside the pore’s lumen. The cysteine residue at amino acid 242 of *VvVDAC4* is marked in blue.

The predicted 3D structure of the VDAC family of *V. vinifera* is shown in Figure 1. VDAC proteins are generally rich in β-sheets (Abrecht et al., 2000; Colombini, 2009). The predicted 3D structures of VvVDAC1, VvVDAC3, VvVDAC4, and VvVDAC5 resemble those of mammalian VDAC1 and zebrafish VDAC2 with 19 transmembrane β-sheets forming β-barrels, and N-terminals forming internal α-helices (Messina et al., 2012; Schredelseker et al., 2014). VvVDAC1 has N-terminals with internal α-helices, but only 16 and 9 transmembrane β-sheets structures, respectively. The 3D structures of VvVDAC6.1 and VvVDAC6.2 lacked the N-terminal α-helices but resembled β-barrels. Among them, VvVDAC2 did not have complete structure, the issues could be due to problems with the available sequence (Figure 1A).

Based on the descriptions of structure from VDAC members, eukaryotic organisms contain 23 conservative amino acid MPS in their *C*-terminal regions: [YH]-X(2)-D-[SPCAD]-X-[STA]-X(3)-[TAG]-[KR]-[LIVMF]-[DNSTA]-[DNS]-X(4)-[GSTAN]-[LIVMA]-X-[LIVMY], X represents any amino acid (Tateda et al., 2011). We analyzed the sequence of MPS domain of VvVDACs from *Vitis*, several plant species and mouse (Supplementary Figure 2). As previous mentioned, the results here showed that *Arabidopsis* AtVDAC1 and AtVDAC3 contained the conserved MPS motif in the *C*-terminal region (Tateda et al., 2009), whereas AtVDAC2 and AtVDAC4 do not. *Vitis* VvVDAC3 and VvVDAC5 also carried a conserved MPS structure domain. In addition, VvVDAC4 has a similar MPS domain but differs by one amino acid residue at its last position 242. Other VvVDACs, including VvVDAC1 and VvVDAC6, which are similar with AtVDAC2, have divergent MPS sequences. Importantly, VvVDAC2 did not contain MPS domain, which suggests that the sequence of VvVDAC2 might not be the full length of VDAC gene and we excluded it out in the following experiments.

The 3D model (Figure 1B) superimposes very well on the structure of VvVDAC3, VvVDAC4, and VvVDAC5 protein containing conserved MPS domain (side and vertical views). The MPS motifs are shown in yellow, while the N-terminals with α-helix are displayed in red. The cysteine residue at amino acid 242 described in VvVDAC4 is marked in blue.

### Phylogenetic Analysis of VDAC in *V. vinifera* and Other Plants

**FIGURE 2:**
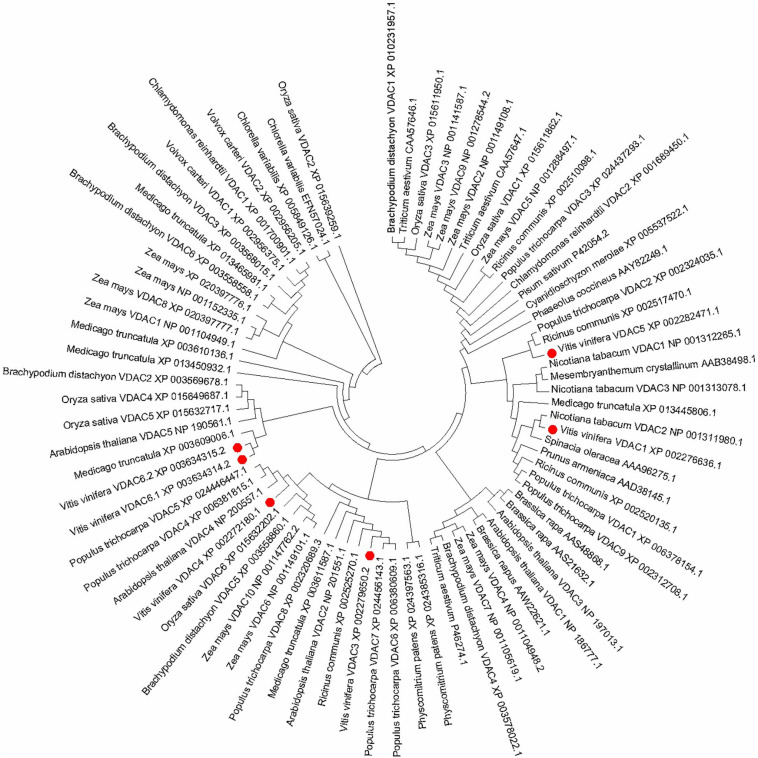
Phylogenetic relationship tree of *VDAC* genes in plants. The phylogenetic tree was constructed in MEGA 7.0 using the maximum likelihood method (1000 bootstrap).

The phylogenetic relationships between from grapevine and other plants are depicted in Figure 2. The unrooted phylogenetic tree indicates that the *VvVDACs* clusters into main five distinct groups. In particular, *VvVDAC1* is closely related to tobacco *NtVDAC3*. *VvVDAC3* belongs to the same cluster and is closely related to *Arabidopsis AtVDAC2* and *AtVDAC5*. *VvVDAC4*, *VvVDAC5*, and *VvVDAC6* are grouped into three clusters and are separate from the other *VDACs*, indicating their complexity and sequence diversity. The two *VvVDAC6* splice variants *VvVDAC 6.1* and *VvVDAC6.2* shared very high similarity with 91.70%.

### Expression Patterns of VvVDAC Genes in Specific Organs in Downy Mildew-Susceptible *V. vinifera*

**FIGURE 3:**
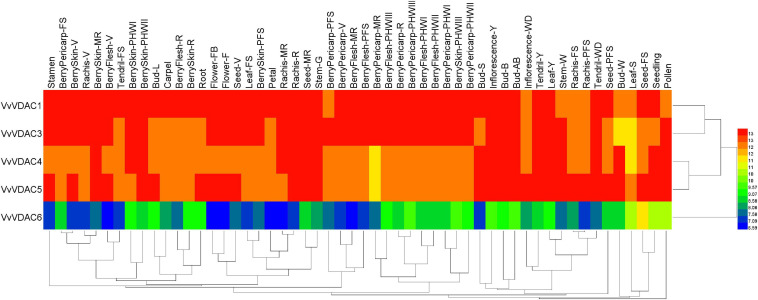
Expression profiles of the *VvVDAC* genes in the tissues/organs of grapevine during the developmental stages. Data were normalized based on the mean expression value of each gene in all tissues analyzed. Red and green boxes indicate high and low expression levels, respectively. BerryPericarp-FS, berry pericarp fruit set; BerryPericarp-PFS, berry pericarp post-fruit set; BerryPericarp-V, berry pericarp véraison; BerryPericarp-MR, berry pericarp mid-ripening; BerryPericarp-R, berry pericarp ripening; Bud-S, bud swell; Bud-B, bud burst (green tip); Bud-AB, bud after burst (rosette of leaf tips visible); Bud-L, latent bud; Bud-W, winter bud; BerryFlesh-PFS, berry flesh post fruit set; BerryFlesh-V, berry flesh véraison; BerryFlesh-MR, berry flesh mid-ripening; BerryFlesh-R, berry flesh ripening; BerryFlesh-PHWI, berry flesh post-harvest withering I (1st month); BerryFlesh-PHWII, berry flesh post-harvest withering II (2nd month); BerryFlesh-PHWIII, berry flesh post-harvest withering III (3rd month); Inflorescence-Y, young inflorescence; Inflorescence-WD, well-developed inflorescence; Flower-FB, flowering begins; Flower-F, flowering; Leaf-Y, young leaf; Leaf-FS, mature leaf; Leaf-S, senescing leaf; BerryPericarp-PHWI, berry pericarp post-harvest withering I (1st month); BerryPericarp-PHWII, berry pericarp post-harvest withering II (2nd month); BerryPericarp-PHWIII, berry pericarp post-harvest withering III (3rd month); Rachis-FS, rachis fruit set; Rachis-PFS, rachis post-fruit set; Rachis-V, rachis véraison; Rachis-MR, rachis mid-ripening; Rachis-R, rachis ripening; Seed-V, seed véraison; Seed-MR, seed mid-ripening; Seed-FS, seed fruit set; Seed-PFS, seed post-fruit set; BerrySkin-PFS, berry skin post-fruit set; BerrySkin-V, berry skin véraison; BerrySkin-MR, berry skin mid-ripening; BerrySkin-R, berry skin ripening; BerrySkin-PHWI, berry skin post-harvest withering I (1st month); BerrySkin-PHWII, berry skin post-harvest withering II (2nd month); BerrySkin-PHWIII, berry skin post-harvest withering III (3rd month); Stem-G, green stem; Stem-W, woody stem; Tendril-Y, young tendril; Tendril-WD, well-developed tendril; Tendril-FS, mature tendril.

The expression patterns of all six *VvVDAC* genes involved in grapevine development were analyzed in the *V. vinifera* cv. Corvina global gene expression atlas from the GEO Datasets (GSE36128), with 54 organs and tissues at different developmental stages analyzed by RNA-sequencing based on previous reports (Fasoli et al., 2012). As shown in Figure 3, *VvVDAC* genes were expressed in numerous grapevine tissues and organs during the growth and developmental stages. *VvVDAC1* exhibited similar and high expression profiles in nearly all tissues and organs. *VvVDAC6* had either moderate or weak expression abundance in all selected tissues and organs, and only showed higher transcript accumulation in the seed fruit set stage, suggesting its limited response in grapevine. *VvVDAC4* and *VvVDAC5* were expressed across all tissues but displayed a typically lower expression in comparison with *VvVDAC1*, which had higher expression in buds and pollen than in other tissues, suggesting their participation in the functioning of buds and pollen. Moreover, *VvVDAC3* maintained a relative higher abundance in leaves and all berry tissues as the berries developed from fruit set stage to berry pericarp post-harvest withering III (3rd month) stage, which suggests that *VvVDAC* might have functions in berry and leaves development. This divergent distribution implies the functional divergence of *VvVDAC* genes for grapevine development.

### Expression Pattern Analysis of VDAC Family in Downy Mildew-Resistant *V. piasezkii*

**FIGURE 4:**
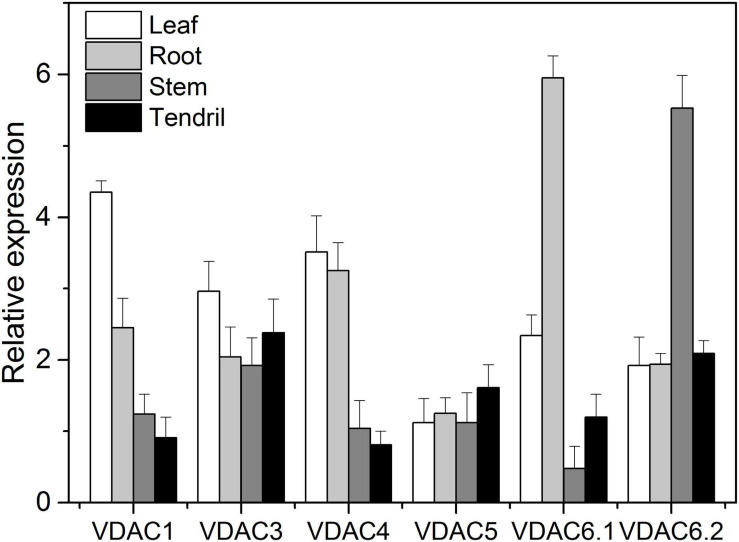
*VpVDACs* expression in grape tissues. Relative *VpVDAC* expression in the leaves, roots, stems, and tendrils of “Liuba-8” (*V. piasezkii*). Error bars indicate the SE (*n* = 3) from three independent biological replicates.

The *VpVDAC* genes expression levels in *V. piasezkii* “Liuba-8” leaves, roots, stems, and tendrils are shown in Figure 4. The expression levels of *VpVDAC1*, *VpVDAC3*, and *VpVDAC4* were higher in the leaves than in the other organs. The expression level of *VpVDAC6.1* was highest in the roots and ≥50% greater than those in the other organs. In the stems, however, only *VpVDAC6.2* was highly expressed. All other *VpVDACs* were expressed at very low levels. In the tendrils, *VpVDAC3* and *VpVDAC6.2* were highly expressed. *VpVDAC5* was also upregulated in the tendrils whereas it was relatively downregulated in the other organs. *VpVDAC3* showed higher abundance in the leaves than in the roots, stems, and tendrils, suggesting that it might play important role in leaf development.

### H_2_O_2_ Accumulation in Transient *VpVDAC3*-Transformed *N. benthamiana* Leaves

Hydrogen peroxide (H_2_O_2_) is an important signaling molecule that initiates transcriptional responses in land plants under biotic and abiotic stress. *VpVDAC1* and *VpVDAC3* overexpression promoted H_2_O_2_ accumulation relative to the empty vector. *VpVDAC3* presented a higher H_2_O_2_ content than *VpVDAC1*. Negligible H_2_O_2_ accumulation was detected in the other *VpVDA*C infiltration area (Supplementary Figure 3).

### Grapevine *VDAC3* Expression in Response to Downy Mildew *P. viticola*

**FIGURE 5:**
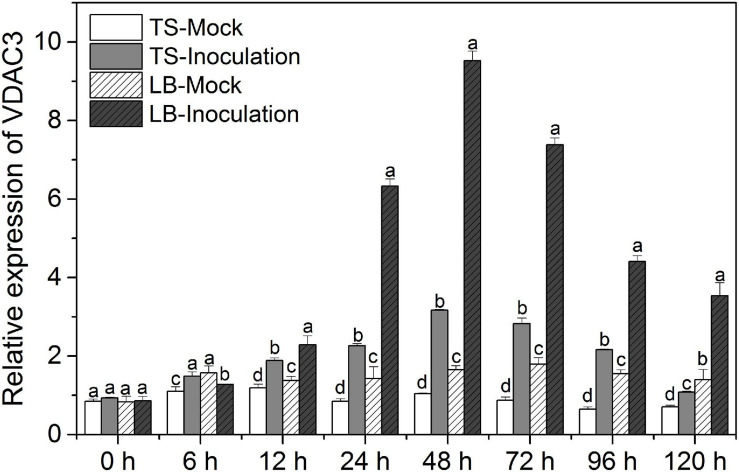
Grapevine *VDAC3* responses to *P. viticola*. Leaves of downy mildew-susceptible “Thompson Seedless” (*V. vinifera*) and downy mildew-resistant “Liuba-8” (*V. piasezkii*) were inoculated either with *P*. *viticola* or water suspension (mock). TS Mock, “Thompson Seedless” sprayed with water suspension; TS Inoculation, “Thompson Seedless” sprayed with *P*. *viticola*; LB Mock, “Liuba-8” sprayed with water suspension; LB Inoculation, “Liuba-8” sprayed with *P*. *viticola*. Samples were harvested for qRT-PCR analysis after 0, 6, 12, 24, 48, 72, 96, and 120 h treatment. Error bars indicate the SE (*n* = 3) from three independent biological replicates. Different letters (a–d) in the same time represent significant difference at *P* < 0.05 level.

Figure 5 shows the temporal *VDAC3* expression patterns in *V. vinifera* cv. “Thompson Seedless” susceptible to downy mildew (*P. viticola*) and Chinese wild grape *V. piasezkii* “Biuba-8,” which is resistant to this pathogen. We sprayed the leaves either with a mock solution or a *P. viticola* sporangial suspension. After inoculation, the *VDAC3* transcript levels were substantially increased in both “Thompson Seedless” and “Liuba-8” and reached a peak at 48 hpi (Figure 5). Meanwhile, grapevine *VDAC3* expression was considerably higher in the resistant “Liuba-8” than it was in the susceptible “Thompson Seedless.” Therefore, we speculated *VpVDAC3* might contribute to stronger resistance in Chinese wild grape *V. piasezkii* “Biuba-8.”

### *VpVDAC3* Exhibited a Higher Resistance to *P. viticola*

**FIGURE 6:**
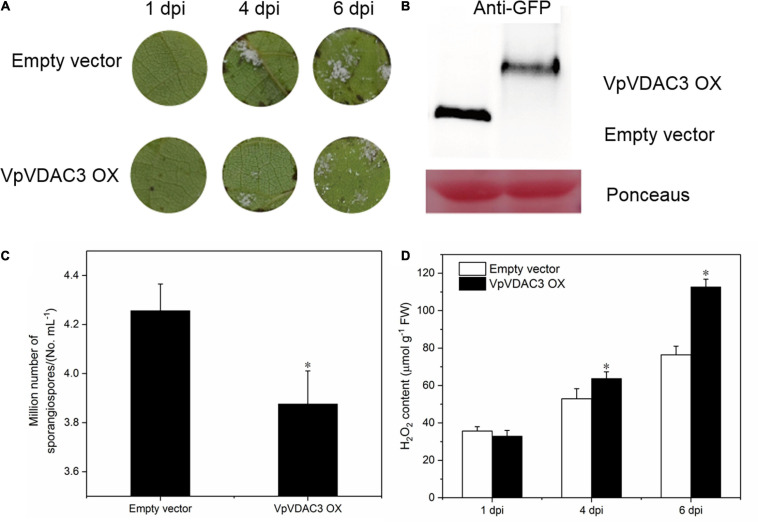
VpVDAC3 enhances H_2_O_2_ accumulation during *P. viticola* infection. **(A)** p35S: VpVDAC3 and the empty vector transiently transformed in *V. vinifera* “Thompson Seedless” with *P. viticola* inoculation. **(B)** Immunoblot detection of protein expression in transiently transformed grapevine leaves after 6 dpi. **(C)** Average sporangia density of *P. viticola* from different genotypes at 6 dpi. **(D)** H_2_O_2_ accumulation. Error bars indicate the SE (*n* = 3) from three independent biological replicates. Asterisks indicate significant difference from the control (empty vector) (*P < 0.05).

To evaluate the role of *VpVDAC3* in response to downy mildew, we transiently overexpressed *VpVDAC3* in the leaves of downy mildew-susceptible “Thompson Seedless” (*V. vinifera*) and checked *P. viticola* disease progression using a leaf disc assay. An empty 35S vector was also transformed as a control. Compared with the control, only a few downy mildew symptoms were observed at 4 days after transformation with *VpVDAC3*; more symptoms were observed with the empty vector (Figure 6A). The functionality of *VpVDAC3* was further validated by agro-mediated transient expression in the leaves with *P. viticola* infection after 6 dpi. *VpVDAC3* transient grapevine leaves were confirmed by protein expression (Figure 6B). Meanwhile, the density and sporangiospore number of downy mildew on the leaf discs of *VpVDAC3* OX were obviously lower than that of the controls (Figure 6C). We further examined the accumulation of H_2_O_2_ between the *VpVDAC3*-overexpressing leaves and the controls at *P. viticola* inoculation sites at 1, 4, and 7 dpi. The results showed that the rate of H_2_O_2_-level rise in the *VpVDAC3*-overexpressing leaves was statistically higher compared to control (Figure 6D).

### Overexpression of *VpVDAC3* Leads to Increased Resistance Against *Pst* DC3000

**FIGURE 7:**
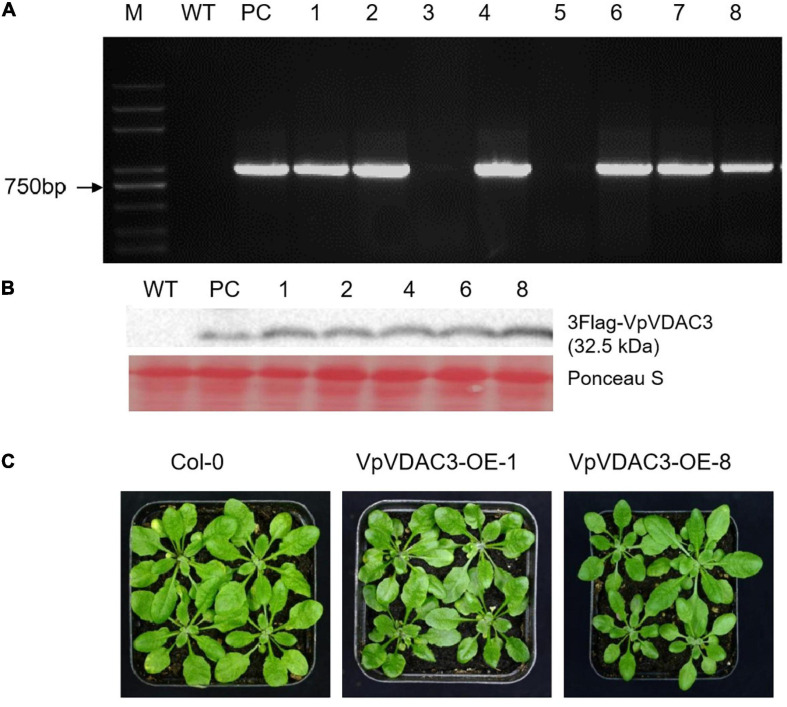
Morphology and *VpVDAC3* expression in transgenic *Arabidopsis* lines. **(A)** RT-qPCR detection of *VpVDAC3* in two independent stable transgenic p35S:VpVDAC3 *Arabidopsis* lines. **(B)** Immunoblot detection of *VpVDAC3* proteins expressed in stable transgenic lines. **(C)** Morphology of WT (Col-0) and two independent transgenic *Arabidopsis* lines grown under normal conditions. M, marker; WT, wild-type Col-0; PC, positive control.

**FIGURE 8:**
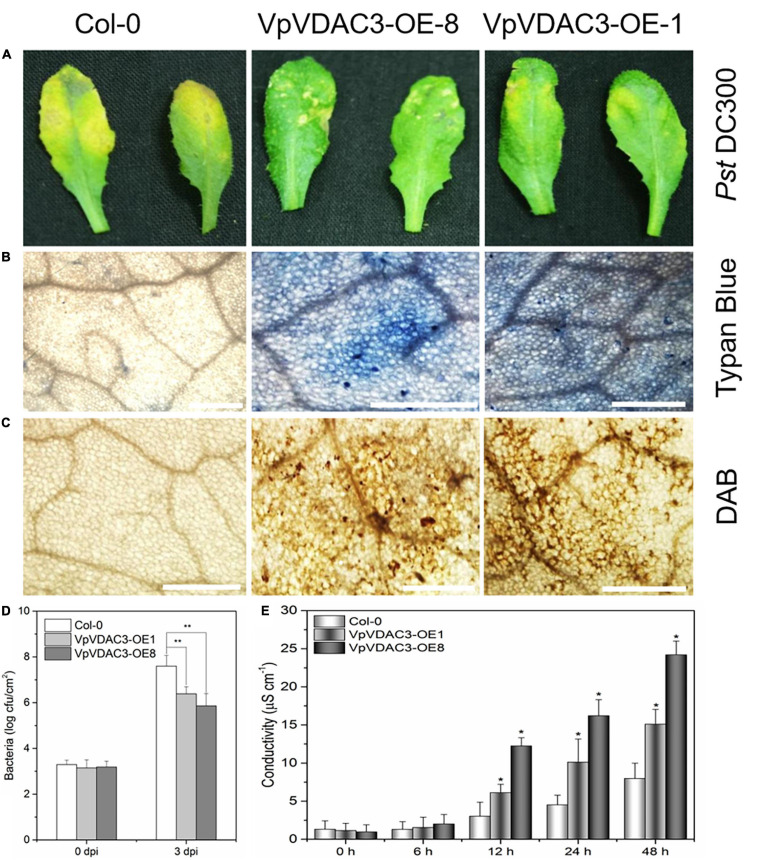
Enhanced *Pst* DC3000 resistance in *VpVDAC3*-OE transgenic *Arabidopsis*. **(A)** Disease symptoms on leaves 72 h after inoculation. **(B)** Micrographs of leaves stained with trypan blue 24 h after infiltration. **(C)** Micrographs of leaves stained with DAB 24 h after inoculation. **(D)** Bacterial growth in leaves inoculated with *Pst* DC3000, Bacterial populations were quantified at 0, and 2 dpi. Vertical bars indicate the standard errors for three independent experiments. **(E)** Ion leakage from leaf tissues inoculated with *Pst* DC3000. Error bars are the SD of three replications results. Error bars indicate the SE (*n* = 3) from three independent biological replicates. An asterisk (*) indicates significant differences compared to the Col-0 line as determined by *t*-tests (*P < 0.05, **P < 0.01) based on three independent replication experiments.

Two stable transgenic lines exhibiting elevated *VpVDAC3* mRNA and protein expression were subjected to further analysis (Figures 7A,B). The transgenic lines were morphologically identical to wild-type (WT) plants under normal growing conditions (Figure 7C). We evaluated the response of these transgenic lines to the *Pst* DC3000, which is a kind of bacterial pathogen (Figure 8). Leaves from *VpVDAC3*-overexpressing and WT (Col-0) *Arabidopsis* were inoculated with *Pst* DC3000. Water-soaked spots and severe chlorosis were visible in Col-0 and the leaves exhibited yellowing after 3 days of inoculation (3 dpi). However, the *VpVDAC3* overexpressing lines displayed slight chlorosis and little yellowing and were healthier at examined time points, suggesting that *VpVDAC3* ectopic expression had greater *Pst* DC3000 resistance than Col-0 (Figure 8A).

Programmed cell death is a HR that is closely related to plant disease resistance, which was visualized using trypan blue, a vital stain that selectively accumulates in dead cells turning them blue (van Wees, 2008). To investigate the capacity of *VpVDAC3* ectopic expression cells to recognize and respond to pathogenic bacteria *Pst* DC3000, trypan blue staining was used. Figure 8B showed more necrotic cells in the transgenic lines overexpressing *VpVDAC3* compared to the controls after *Pst* DC3000 infection. ROS levels are also closely associated with a pathogen infection. We also used ROS reactive dyes histochemical 3,3′-diaminobenzidine (DAB) to test H_2_O_2_ contents in infected rosette leaves. H_2_O_2_ is visualized as reddish brown stain formed by the reaction of DAB with the endogenous H_2_O_2_. The results showed that *VpVDAC3* transgenic leaves stained strongly and accumulated more H_2_O_2_ than the WT (Figure 8C), demonstrating that *VpVDAC3* could enhance resistance to *Pst* DC3000. Less bacterial growth were also observed in *VpVDAC3* ectopic expression leaves and which was corrected with stronger resistance (Figure 8D). The leaf conductivity represents the extent of membrane injury. Transgenic plants infected with *Pst* DC3000 demonstrated significantly higher electrolyte release responses and greater leakage than Col-0 (Figure 8E).

**FIGURE 9:**
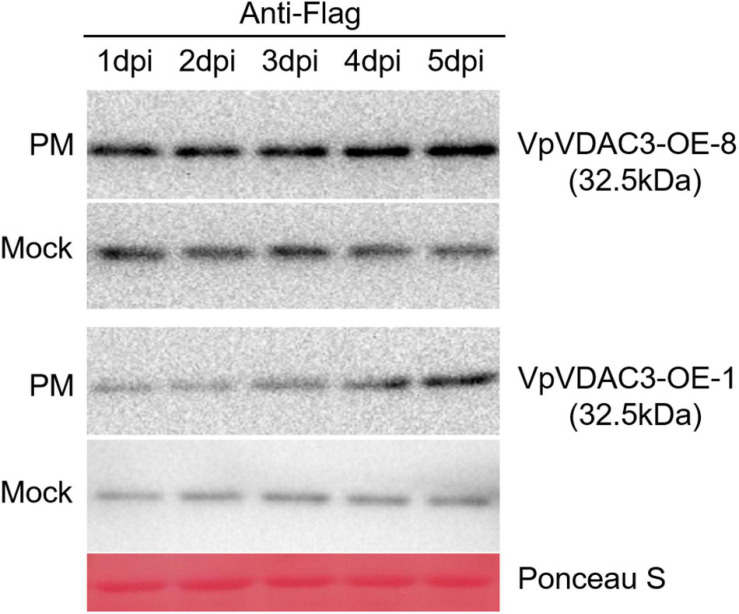
*VpVDAC3* protein accumulation during *Pst* DC3000 infection. Western blot analysis of two stable p35S:VpVDAC3 transgenic *Arabidopsis* lines (*VpVDAC*-OE-3 and *VpVDAC*-OE-8) after *Pst* DC3000 inoculation. Protein samples were extracted after 1, 2, 3, 4, and 5 dpi. Data are representative of three independent experiments.

Expression of VpVDAC3 protein levels during *Pst* DC3000 infection are confirmed in Figure 9. The protein levels in mock-treated leaves did not significantly differ at various time points. However, the VpVDAC3 protein level accumulated after *Pst* DC3000 infection, especially at time points 5 dpi. Thus, VpVDAC3 protein was stabilized during pathogen infection and involved in the increased-resistance in response to pathogen. Together, our results support the idea that VpVDAC3 plays a role in increasing plant resistance against *Pst* DC3000 through protein post-transcriptional regulation like preventing accumulation after *Pst* DC3000 pathogen infection.

## Discussion

Voltage-dependent anion channels are the most abundant proteins in the outer mitochondrial membrane. The latter regulates the movement of small mitochondrial and cytoplasmic molecular metabolites and controls other physiological functions (Blachly-Dyson and Forte, 2001; Noskov et al., 2016). Previous studies on VDACs focused mainly on their functions in mammalian cells. The number of VDACs is generally higher in plant cells than mammalian cells (Takahashi and Tateda, 2013). Nevertheless, VDAC functions might be more diverse in the latter. Moreover, there are few studies regarding the importance of VDACs in plant pathogens and stress resistance, and more detailed research in these areas is still required. The present study mainly explored the expression patterns and potential pathogen signaling roles of *VDAC*s in grapevine.

A previous study showed that there are three VDAC protein isoforms in mammals (Messina et al., 2012). Then plant VDACs were also constantly identified. Three *VDAC*s were identified in tobacco (*Nicotiana tabacum*) (Tateda et al., 2009), five in *Lotus japonicus*, five in *Arabidopsis* (Tateda et al., 2011), and eight in rice (*Oryza sativa*) (Xu et al., 2015). Here, we showed that grape (*V. vinifera*) has five *VDAC* isoforms and two variants. Our molecular phylogenetic analysis disclosed that *VvVDACs* belong to different clusters and these duplications may indicate that they play various functions in grapevine. Evolutionary analysis of *VDAC* sequences in plants, animals, and fungi revealed that *VDAC* polygenic families formed by *VDAC* replication in the genome may occur independently after differentiation (Homblé et al., 2012). In *Arabidopsis*, VDACs responded to cold, drought, salinity, and pathogens (Lee et al., 2009), and the two of them also have been reported to exhibit the effect of plant growth, the *atvdac1* mutant exhibited faster germination kinetics (Li et al., 2013) and the *atvdac2* mutant produced an abscisic acid (ABA)-insensitive phenotype (Yan et al., 2009). Therefore, we assumed that the characterized various VDAC isoforms might play roles in several serious grapevine diseases like *Erysiphe necator*, *P. viticola*, *Botrytis cinerea*, or have effects on grape development such as berry size and color. Related research need to do further studies to understand and parse their functions.

Sequence similarity alignments of animals, fungi, and plants demonstrated that VDAC proteins exhibited low sequence identity. However, the sequences still shared a few common features. Each one had a MPS motif near the *C*-terminal region and a β-signal motif at the *C*-terminal of the protein (Homblé et al., 2012). We discovered that grapevine VDAC3, VDAC4, and VDAC5 each contained a conserved MPS motif that were also reported for 30 other eukaryotic VDAC sequences. In this study, however, VvVDAC1 and VvVDAC6 carried a divergent MPS motif. Young et al. (2007) stated that the conserved MPS motif is absent in certain VDAC sequences. Takahashi and Tateda (2013) analyzed MPS motif conservation in the VDAC sequences of 75 different dicots and divided them into two groups. One displayed a highly conserved MPS motif while the other presented with a high divergence rate. Grapevine *VvVDAC3*, *VvVDAC4*, and *VvVDAC5* might belong to the former group whereas *VvVDAC1* and *VvVDAC6* could be in the latter.

In addition, the research on the MPS of VDACs from other plants species exhibited that VDACs contained a canonical MPS motif are exclusively localized to mitochondria, while the divergent ones are localized not only in mitochondria but also in other unidentified cellular regions. *Arabidopsis* VDAC1 and VDAC3 with well-conserved MPS motif were detected primarily in mitochondria (Tateda et al., 2011). Further analysis the cellular localization of *Pisum sativum* and *L. japonicus* VDAC isoforms carrying a conserved MPS motif showed the similar situation as *Arabidopsis* (Clausen et al., 2004; Wandrey et al., 2004). However, VDACs bear the divergent MPS motif in the region present additionally in numerous small vesicles at the cell periphery (Wandrey et al., 2004). Further, Tateda et al. (2011) mutated the conserved MPS motif of AtVDAC1 and found that the subcellular localization of AtVDAC1-m2 exhibited the same as that of AtVDAC2 (containing a divergent MPS), indicating that MPS motif is a key domain functioning to restrict VDAC localization to mitochondria.

Voltage-dependent anion channel protein amino acid sequences were not conserved among different species. Nevertheless, the structural framework of VDAC protein may be relatively conservative as the positions and/or lengths of the β-sheets and the α-helices were conserved in all species studied (Young et al., 2007). The four *VvVDACs* contained 19 β-sheets and α-helices at the *N*-terminal region in the sequence. Similar basic properties were reported for the predicted structures of mouse and human VDAC1 and plant VDACs (Homblé et al., 2012). Though VDAC proteins have diverged during evolution, their protein structure was conserved. Therefore, the motif has common and important roles among various kingdoms. However, its functional significance remains to be determined. The present work lays the foundation for future investigations into the relationship between the structure and special functions of VDAC protein.

Previous reports have shown that the levels of VDAC genes expression in various plant tissues are different (Elkeles et al., 1995; Al Bitar et al., 2003). Furthermore, gene/protein function may become specialized *via* changes in the gene expression pattern. We analyzed the expression profile of *VDACs* in various tissues and organs in downy mildew-susceptible “Thompson Seedless” (*V. vinifera*) and downy mildew-resistant “Liuba-8” (*V. piasezkii*). The variant expression patterns observed for each *VDAC* suggest that genome redundancy partially accounts for the observed duplication and higher *VDAC* copy number in plants. In addition, *VpVDAC3* was expressed in all plant parts whereas it displayed a peak transcript abundance in some tissues, like, for instance, the leaves and berries during the developmental stages, indicating its important role in these tissues and organs’ functions. The different organ-specific expression pattern was also reported for *VDACs* in developing wheat and rice (Elkeles et al., 1995; Xu et al., 2015). In addition, some *VvVDACs* only displayed high expression in specific tissues, indicating that it might have a unique function in specific developmental stages. Taken together, most *VvVDACs* were expressed in most tissues and most developmental stages, which reveals that *VvVDAC* genes play multiple important roles in various developmental and biological processes.

Reactive oxygen species (ROS) such as hydrogen peroxide (H_2_O_2_) are destructive molecules formed in the cell by oxidative metabolism (Gough and Cotter, 2011). Here, we used a transient assay on *N. benthamiana* leaves to overexpress *VpVDACs* and examine their roles in the oxidative burst. When *VpVDAC3* was upregulated, mitochondrial ROS production and accumulation increased (Supplementary Figure 3). Earlier studies showed that *VDAC* is vital to mitochondrial physiology and affects ROS production (Veenman et al., 2008). *VpVDAC3* overexpressing *Arabidopsis* plants had a stronger host defense response to *Pst* DC3000 infection than WT plants. Similar results were obtained for detection in transient grapevine leaves after *P. viticola* infection correlating with H_2_O_2_ accumulation detected in leaves overexpressing *VpVDAC3*. However, the mechanism by which *VDACs* regulate H_2_O_2_ production remained unknown. The roles of animal *VDACs* in ROS homeostasis are well documented (Reina et al., 2010). In contrast, little is known about the functions of plant *VDACs* in ROS regulation. To clarify the mechanism of *VpVDAC3* response to *Pst* DC3000, we evaluated the changes in VpVDAC3 protein level following pathogenesis. The VpVDAC3 protein level were higher after *Pst* DC3000 infection (Figure 9), which might be caused by the protein post-transcriptional modification when response to pathogen. A previous study showed that *Arabidopsis AtVDAC1–AtVDAC4* were upregulated following *Pst* DC3000 inoculation (Tateda et al., 2009). Another study showed that *Pst* DC3000 challenge upregulated *VDACs* (Lee et al., 2009). A subsequent report confirmed that both virulent *Pst* DC3000 and avirulent *Pst* DC3000 avrRpt2 upregulated *VDACs* (Tateda et al., 2011).

Changes in *VDAC* expression were documented in response to biotic stress such as a HR to pathogen attack (Lee et al., 2009). To establish the role of *VpVDAC3* in biotic stress response, we investigated grapevine resistance to the highly destructive downy mildew caused by *P. viticola* (Kennelly et al., 2007). We found that *VpVDAC3* participated in downy mildew resistance and was strongly expressed in the *P. viticola*-resistant *V. piasezkii* “Biuba-8.” Transient overexpression of *VpVDAC3* in grapevine leaves increased the plant’s resistance to *P. viticola* infection and led to greater accumulation of H_2_O_2_ content. Our previous study indicated that *VpVDAC3* plays important roles in the biological functions of grapevine. *VDAC3* from *V. piasezkii* “Biuba-8” interacted with PR10.1 and triggered pathogen resistance (Ma et al., 2018), which is similar with *Arabidopsis VDAC1*, contributes to disease resistance by regulating the production of H_2_O_2_, which is also implicated in plant disease resistance (Tateda et al., 2011). Meanwhile, the expression of grapevine *VvVDAC3* in the susceptible “Thompson Seedless” can also be induced slightly by *P. viticola*. Therefore, we cannot excluded that *VvVDAC3* might also contribute to some resistance in *V. vinifera.* The foregoing studies have helped elucidate the functions of *VDACs* response to pathogens, providing insight into how regulation pathways may have been adapted to improve the resistance to downy mildew pathogen stress in grapevine.

## Conclusions

In conclusion, the findings of this study demonstrate that grapevine *VDAC3* could enhance the tolerance of downy mildew pathogen stress in grapevine correlating with H_2_O_2_ accumulation. And during the process, the transcript level of *VpVDAC3* was induced highly and the protein level was accumulated by post-transcriptional regulation. The results are expected to provide more sustainable production systems for viticulture and new strategies of protection against downy mildew in grapevine through the development of a resistant grapevine germplasm, which in turn could lead to the discovery of new genetic resources and the diversification of the regulatory mechanisms of resistance genes.

## Data Availability Statement

The original contributions presented in the study are included in the article/Supplementary Material, further inquiries can be directed to the corresponding author/s.

## Author Contributions

YX conceived and designed the experiments. TX, XW, HM, and LS performed the experiments. TX, XW, HM, and WW analyzed the data. TX, JM, and XW wrote the manuscript. All authors participated in this research and approved the manuscript.

## Conflict of Interest

The authors declare that the research was conducted in the absence of any commercial or financial relationships that could be construed as a potential conflict of interest.

## Footnotes

1 http://gsds.cbi.pku.edu.cn/

2 https://www.ncbi.nlm.nih.gov/geo/query/acc.cgi?acc=GSE36128
