# Supplementary material for: Functional Characterization of VDACs in Grape and Its Putative Role in Response to Pathogen Stress
Source: Front Plant Sci. 2021 Jun 16;12:670505. doi: 10.3389/fpls.2021.670505 (PMC8242593; doi:10.3389/fpls.2021.670505)
Supplement: Supplementary file 1 [file Data_Sheet_1.docx]

Supplementary Material

## Supplementary Figures


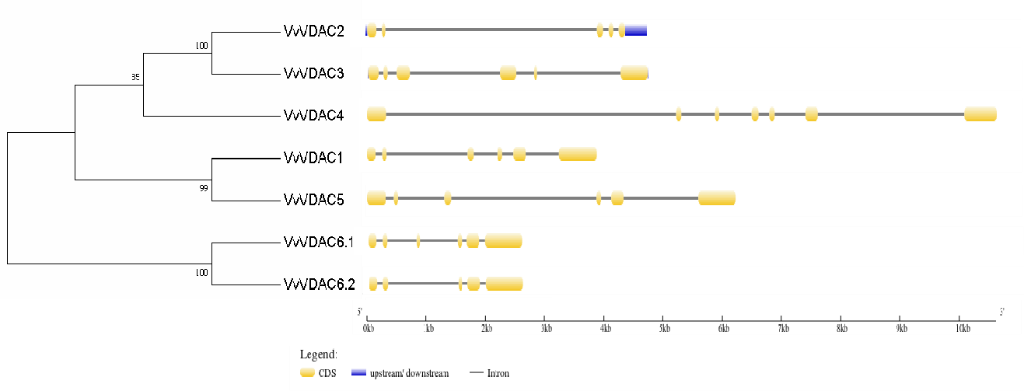


**Supplementary Figure 1.** Phylogenetic tree and gene structure of grape VDACs. Phylogenetic relationship tree of *VDAC* genes in grapevine. The phylogenetic tree was constructed in MEGA 7.0 using the Neighbor-Joining Method (1000 bootstrap). Intron-exon structure of VvVDAC genes was explored using GSDS. The blue boxes, yellow boxes and black lines represented UTR, exons and introns, respectively. The scale of gene length are presented at the bottom.

**
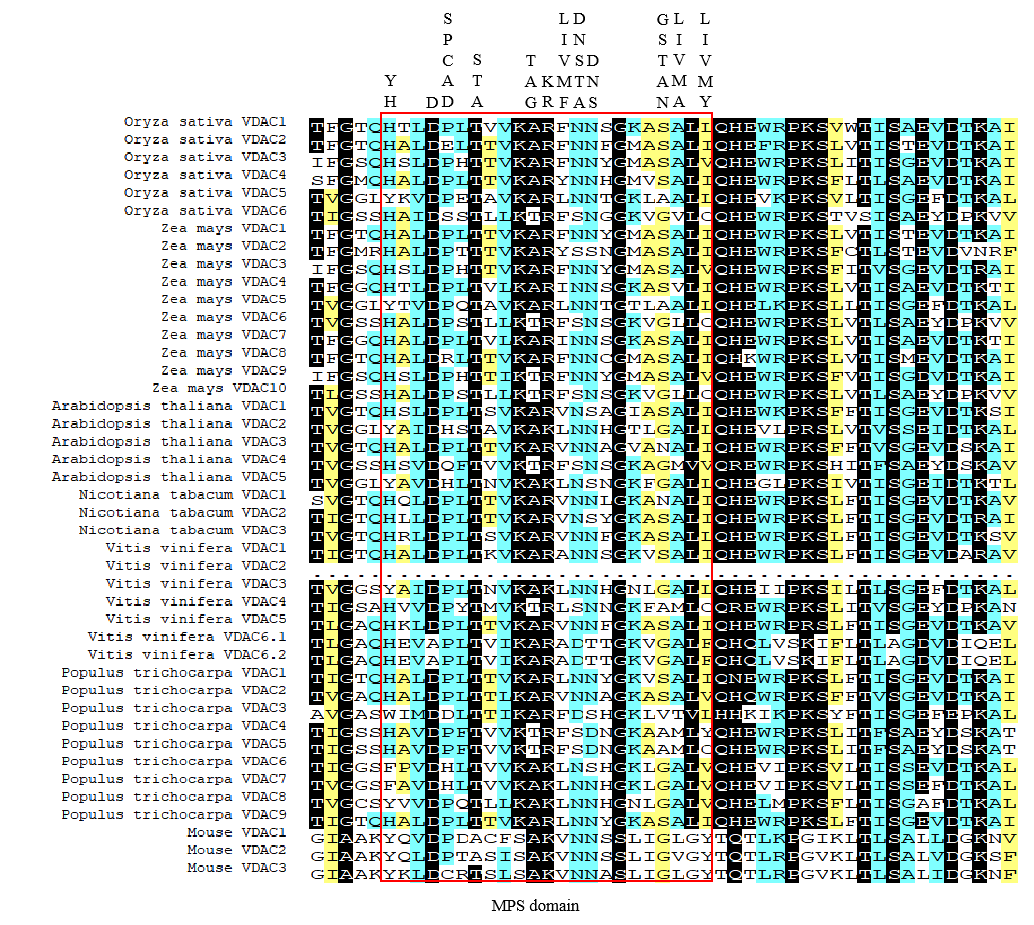
**

**Supplementary Figure 2.** Amino acid sequence alignment of the MPS motif regions in VDACs. The alternative residues of the MPS motif are displayed from plants to mammal above the amino acid sequences that were aligned by the DNAMAN 9.0 software. Identical and similar residues are highlighted. The MPS motifs are indicated by red square bracket. The accession numbers used were mouse VDAC1 (NP_001349622.1), mouse VDAC2 (NP_035825.1), mouse VDAC3 (NP_001185927.1). other accession numbers were shown in Figure 2.


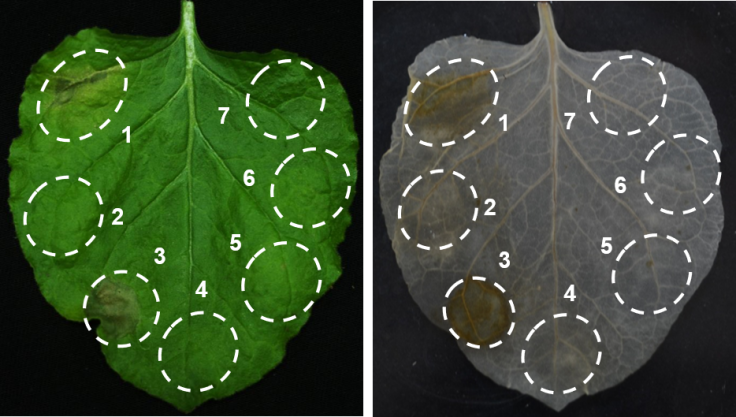


**A**

**B**

**Supplementary Figure 3.** *VvVDAC3* promotes H_2_O_2_ accumulation. (A) Transient tobacco leaves (B) Transient tobacco leaves after histochemical staining with DAB. Five *VDAC* from *V. piasezkii* were transiently expressed in tobacco leaves, which were then stained with DAB 72 h after inoculation. (1) Bax triggered cell death and was used as a positive control. (2) *VpVDAC1;* (3) *VpVDAC3*; (4) *VpVDAC4*; (5) *VpVDAC5*; (6) *VpVDAC6*.*1*; (7) empty vector.

## Supplementary Tables

**Supplementary Table 1.** List of primers used for qRT-PCR.

| **Name** | **Sequence (5’-3’)** | **Purpose** |
| --- | --- | --- |
| VDAC1-F | ACGACAATTACTGTTGATGAACCTGC | qRT-PCR |
| VDAC1-R | GTTCCCAGTGGCAGTGTCAAAC | qRT-PCR |
| VDAC3-F | GGCAGGTTATGATACTACATCTGG | qRT-PCR |
| VDAC3-R | GGTCAATTGCATAAGACCCTCCT | qRT-PCR |
| VDAC4-F | CCTTATGGTGGAACTACAGTTGC | qRT-PCR |
| VDAC4-R | AGCTCCATTGTTAGCCTTCGGG | qRT-PCR |
| VDAC5-F | GCTGCTCTCCTACCGGAGT | qRT-PCR |
| VDAC5-R | GAAGTTAACAATGGGATTCGCTGTC | qRT-PCR |
| VDAC6.1-F | CTCAAATTTGTCGGTCGACTTCG | qRT-PCR |
| VDAC6.1-R | GTCAACGAAGCAGACACGATTGG | qRT-PCR |
| VDAC6.2-F | TGGCCTTGAGATTGGGGGAG | qRT-PCR |
| VDAC6.2-R | GCAGTATCCCCCTTGTCTTCC | qRT-PCR |
| VvActin-F | CCATCCTTCGTCTTGACCTTGCTG | qRT-PCR |
| VvActin-R | AGTGGTGAACATGTAACCCCTCTC | qRT-PCR |

**Supplementary Table 2.** Characterization of *VDAC* members in the *V. vinifera* genome

| Gene name | Accession number | Location | CDS (bp) | Peptide (aa) |
| --- | --- | --- | --- | --- |
| VvVDAC1 | XM_002276600.3 | chr1:10815279..10818995 | 831 | 276 |
| VvVDAC2 | XM_003632453.1 | chr7:15426943..15432003 | 429 | 142 |
| VvVDAC3 | XM_002279614.4 | chr7:15426943..15432003 | 831 | 276 |
| VvVDAC4 | XM_002272144.3 | chr11:18708857..18714197 | 834 | 277 |
| VvVDAC5 | XM_002282435.3 | chr14:28844435..28850458 | 831 | 276 |
| VvVDAC6.1 | XM_003634266.3 | chr17:6167654..6170135 | 777 | 258 |
| VvVDAC6.2 | XM_003634267.3 | chr17:6167654..6170135 | 723 | 240 |

**Supplementary Table 3.** Sequence identity of grape VDACs.

| Protein | VvVDAC1 | VvVDAC2 | VvVDAC3 | VvVDAC4 | VvVDAC5 | VvVDAC6.1 | VvVDAC6.2 |
| --- | --- | --- | --- | --- | --- | --- | --- |
| VvVDAC1 | 100.00% |  |  |  |  |  |  |
| VvVDAC2 | 27.5% | 100.00% |  |  |  |  |  |
| VvVDAC3 | 51.40% | 50.00% | 100.00% |  |  |  |  |
| VvVDAC4 | 49.50% | 23.80% | 45.50% | 100.00% |  |  |  |
| VvVDAC5 | 78.60% | 26.40% | 51.80% | 47.30% | 100.00% |  |  |
| VvVDAC6.1 | 40.10% | 7.70% | 25.80% | 26.60% | 38.80% | 100.00% |  |
| VvVDAC6.2 | 36.60% | 7.60% | 26.40% | 24.90% | 35.60% | 91.70% | 100.00% |
